# *Erratum*: Vol. 65, No. SS-12

**DOI:** 10.15585/mmwr.mm6948a9

**Published:** 2020-12-04

In the Surveillance Summary “Abortion Surveillance — United States, 2013,” data on the number of known previous induced abortions for women having abortions in this reporting year were erroneously included for New York City. These estimates did not meet reporting standards and should have been excluded from this report. When corrected, among women with abortions in the reporting year, the proportion with no previous induced abortions increased, and the proportion with one or more previous induced abortions decreased. In addition, Nebraska was erroneously excluded as a reporting area for the relevant years of comparison for estimates on reported abortions, by known number of previous induced abortions. Data have been updated to include Nebraska.

On page 9, the last paragraph of the first column should have read “Data from the **38** areas that reported the number of previous abortions for women who obtained abortions in 2013 indicate that the majority (**57.7%**) had no previous abortions, **35.5%** had one to two previous abortions, and **6.8%** had three or more previous abortions (Table 17). Among the 30 reporting areas^§§§§^ that provided data for the relevant years of comparison (2004 to 2013, 2004 versus 2008, 2009 versus 2013, and 2012 versus 2013), the percentage of women who had zero or one to two previous abortions did not change appreciably over time: **57.8%**, **58.2%**, and **58.1%** had zero previous abortions in 2004, 2012, and 2013, respectively, and **35.9%**, **34.9%**, and **35.0%** had one to two previous abortions in 2004, 2012, and 2013, respectively. In contrast, among these 30 areas, the percentage of women who had three or more previous abortions increased from 2004 to 2013 but did not change appreciably from 2012 to 2013: **6.3%** had three or more previous abortions in 2004, as compared with **6.8%** in 2012 and **6.8%** in 2013.” New York City should have been included, and Nebraska should not have been included in the ^§§§§^ footnote, which lists reporting areas that were not included in these estimates.

In Table 17, the line for New York City should be deleted. For the total line, the numbers and percentages should have read **213,976 (57.7), 92,992 (25.1), 38,551 (10.4), 25,291 (6.8), 370,810 (98.7)**. The * footnote should have read “Data from **38** reporting areas; excludes **14** areas (California, Connecticut, District of Columbia, Florida, Georgia, Illinois, Maryland, New Hampshire, New Mexico, **New York City**, New York State, North Carolina, Wisconsin, and Wyoming) that did not report, did not report by the number of previous induced abortions, or did not meet reporting standards.” The total in the ** footnote should have been **375,638**.

